# Compression in Acute Rheumatism

**Published:** 1876-04

**Authors:** 


Compression of the Joints in Acute Rheumatism.—In the *Deutsch. Arch. fur klin Med.,* Dr. F. Riegel reports his results in 41 cases of acute rheumatism in which he resorted to fixation and compression of the affected joints. The principle advantage claimed for this plan consists in the almost immediate relief from pain afforded by it. The joints should be surrounded by cotton and pieces of pasteboard, and the bandage carefully and evenly applied in order to secure uniform pressure.—*Centralblat f. d. med. Wissenschaften No.* 52, 1875.

